# Effects of a Bout of Intense Exercise on Some Executive Functions

**DOI:** 10.3390/ijerph17030898

**Published:** 2020-01-31

**Authors:** Marinella Coco, Andrea Buscemi, Claudia Savia Guerrera, Donatella Di Corrado, Paolo Cavallari, Agata Zappalà, Santo Di Nuovo, Rosalba Parenti, Tiziana Maci, Grazia Razza, Maria Cristina Petralia, Vincenzo Perciavalle, Valentina Perciavalle

**Affiliations:** 1 Department of Biomedical and Biotechnological Sciences, University of Catania, 95123 Catania, Italy; claguerre@hotmail.it (C.S.G.); azappala@unict.it (A.Z.); Parenti@unict.it (R.P.); 2 Motor Activity Research Center (CRAM) University of Catania, 95123 Catania, Italy; 3 Horus Social Cooperative, Department of Research, 97100 Ragusa, Italy; andreabuscemi@virgilio.it; 4 Department of Research, Italian Center Studies of Osteopathy, 95100 Catania, Italy; 5 Department of Human and Social Sciences, School of Sport Sciences, Kore University, 94100 Enna, Italy; didinawoody@gmail.com (D.D.C.); perciava@libero.it (V.P.); 6 Department of Pathophysiology and Transplantation, Human Physiology Section, University of Milan, 20122 Milan, Italy; paolo.cavallari@unimi.it; 7 Department of Educational Sciences, 95100 Catania, Italy; s.dinuovo@unict.it (S.D.N.); m.cristinapetralia@gmail.com (M.C.P.); valentinaperciavalle@hotmail.it (V.P.); 8 Independent Researcher, 95100 Catania, Italy; tizianamaci@libero.it (T.M.); grazia.r@live.it (G.R.)

**Keywords:** executive functions, young sport, blood lactate, exhaustive exercise, fatigue, elderly sport

## Abstract

The present study examined the effects of an exhaustive exercise on executive functions by using the Stroop Color Word Test (SCWT), Trail Making Test (TMT), A and B, and simple Reaction Time (RT). Thirty adults agreed to participate; 15 participants had a mean age of 24.7 years ± 3.2 Standard Deviation (SD, Standard Deviation) (group YOUNG), while the remaining 15 had a mean age of 58.9 years ± 2.6 SD (group OLD). Each subject performed the cognitive tasks at rest and blood lactate was measured (pre); each subject executed the acute exhaustive exercise and, immediately after the conclusion, executed the cognitive tasks and blood lactate was again measured (end). Cognitive tests were repeated and blood lactate measured 15 min after its conclusion of the exhaustive exercise (post). We observed: (1) a significant positive correlation between blood lactate levels and RT levels; (2) a significant negative relationship between levels of blood lactate and the SCWT mean score; (3) no significant correlation between blood lactate levels and TMT scores (time and errors), both A and B; (4) variations in blood lactate levels, due to exhaustive exercise, and parallel deterioration in the execution of RT and SCWT are significantly more pronounced in the group YOUNG than in the group OLD. The present study supports the possibility that high levels of blood lactate induced by an exhaustive exercise could adversely affect the executive functions pertaining to the prefrontal cortex.

## 1. Introduction

The effects of acute physical exercise on the cognitive performances of an adult individual are still under discussion [1,2,3,4,5,6,7,8,9,10,11,12,13,14,15,16,17,18]. The existing literature tends to highlight a positive relationship if the exercise is of sub-maximal intensity, while the effects seem to be negative for exhaustive exercises [19,20,21,22,23,24,25,26]. 

Within cognitive processes, there are few studies on the effects of an exhaustive exercise on executive functions. [27,28,29,30,31,32]. This term indicates a set of cognitive processes that allow us to plan, regulate, control, and evaluate behaviors that are useful for achieving a goal [17]. Executive functions include planning, problem solving, flexibility, inhibition, multitasking, and working memory [18]. A negative effect of high blood lactate levels induced by an exhaustive exercise or with an intravenous infusion of a lactate solution has been found for attentional processes [3,5,6,8,10,19]. Regarding the working memory, a negative effect of exhaustive exercise on both non-spatial working memory and motor working memory was found [9]. Concerning other executive functions, a study that used a combination of a Spatial Delayed-Response task and a Go/No-Go task found no correlation between blood lactate levels and cognitive functions [12].

The purpose of the present study was to examine the effects of an exhaustive exercise on executive functions by using the Stroop Color Word Test (SCWT), correlated with cognitive flexibility and resistance to interference from external stimuli [25], and Trail Making Test (TMT), associated with visual attention and task switching [28]. Simple Reaction Time (RT), as basic measure of processing speed [33], was also evaluated.

## 2. Materials and Methods

### 2.1. Participants

In this study, 30 adults agreed to participate; 15 participants had a mean age of 24.7 years ± 3.2 SD (group YOUNG), while the remaining 15 had a mean age of 58.9 years ± 2.6 SD (group OLD). All participants had practiced amateur sports for at least one year and had medical authorization to practice non-competitive sports. Table 1 illustrates the anthropometric characteristics of the participants. The T-test showed that there were no statistically significant differences in height, weight, and Body Mass Index (BMI).

The study was approved by the Ethical committee of the University of Milan (number 15/16). All participants were informed about the trials of the study and the anonymity of their answers before providing their written consent to participate, in accordance with the Declaration of Helsinki.

### 2.2. Experimental Design

The tests were executed between 9 am and 1 pm, with participants who had eaten breakfast before 8 am [5]. Each subject performed the cognitive tasks at rest and blood lactate was measured (pre). Each subject executed the acute exhaustive exercise and, immediately after the conclusion, performed the cognitive tasks and blood lactate was again measured (end). Finally, cognitive tests were repeated and blood lactate measured 15 min after the exhaustive exercise (post). The overall duration of the cognitive tests did not exceed 6 min.

### 2.3. Exercise

The participants performed a maximal multistage discontinuous incremental cycling test on a mechanically braked cycloergometer (Monark, Sweden), at a pedaling rate of 60 rpm, while an electrocardiogram was monitored. Each subject started with unloaded cycling during 3 min, and the load was increased by 30 W every 3 min until volitional exhaustion or the required pedaling frequency of 60 rpm could not be maintained [17].

### 2.4. Blood Lactate

Blood lactate was measured before as well as at the end and 15 min after the conclusion of the exercise, using a “Lactate Pro 2” portable lactate analyzer (Arkray Inc., Kyoto, Japan), since this automated lactate analyzer has a good reliability [1].

### 2.5. Simple Reaction Time

The procedure for measuring RT was the same one as the one that was used previously [3]. The subject must press the bar-space of the computer to appearing on the screen of the symbol target “star.” This is a RT task that demands an intense simple attention; in order to avoid settling habituation, the target presentation was randomized with intervals comprised between 1 and 3 s.

### 2.6. Stroop Colour Word Test

In the present study the golden version of the SCWT was used [25]. The test comprises three parts. In the first part, the subject reads a list of 50 names printed with black ink. In the second part, the subject observes 50 circles of different colors and must indicate their color. In the third part, the subject receives a list of 50 words with the names of the colors written with an incongruent color ink; the subject must indicate the color of the ink, ignoring the written word. The number of correct answers in 45 s in the third part is considered to be representative of the “interference” component of the SCWT.

### 2.7. Trial Making Test

TMT was chosen for evaluating information processing speed and executive functioning [28]. The TMT consists of two parts. In TMT-A, the subject had to draw lines sequentially connecting 25 numbered circles distributed on a sheet of paper. In TMT-B, the subject must alternate between numbers and letters distributed on the sheet. The score on each part represents the number of seconds required to complete the task and the number of errors.

### 2.8. Statistical Analysis

Data was collected and averaged, and then compared with the paired t test (2-tailed) or 1-way repeated measures analysis of variance (ANOVA; Friedman test), followed by Dunn’s Multiple Comparison Test. Correlation analysis was carried out using one-tailed Pearson’s correlation. Significance was set at *p* < 0.05. All descriptive statistics are reported as mean ± SD. All analyses were performed by using GraphPad Prism version 6.03 for Windows (GraphPad Software, San Diego, CA, USA).

## 3. Results

**Figure 1:**
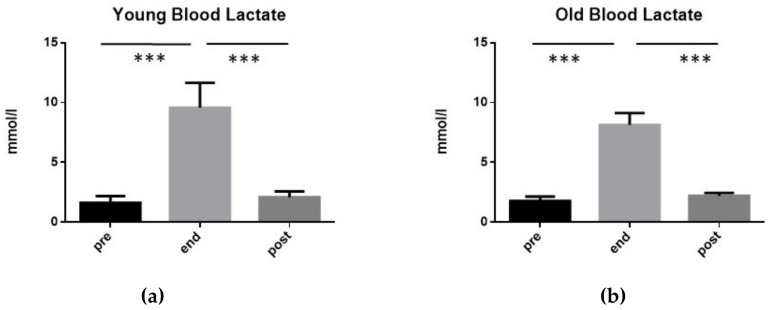
Blood lactate values of the 15 subjects of group YOUNG (**a**) and of the 15 subjects of group OLD (**b**) performing an exhaustive exercise. In both cases, blood lactate mean values measured before the exercise (pre), at its conclusion (end), as well as 15 min after its end (post) are illustrated. Symbols from ANOVA with Dunn’s multiple comparison test: *** *p* < 0.001.

As can be seen in Figure 1, in both YOUNG and OLD groups the blood lactate increased significantly at the end of the exhaustive exercise, and returned to the pre-exercise values 10 min after its end. In particular, in the group YOUNG, blood lactate levels increased from 1.63 mmol/L (±0.57 SD) before the exercise, to 9.58 mmol/L (±2.08 SD) at its end, and returned to pre-exercise values after 15 min (2.1 mmol/L ± 0.48 SD).

However, the level reached by blood lactate at the end of the exercise in the group YOUNG was significantly lower with respect to the value measured in the same moment in the group OLD (t-test: *p* < 0.05).

**Figure 2:**
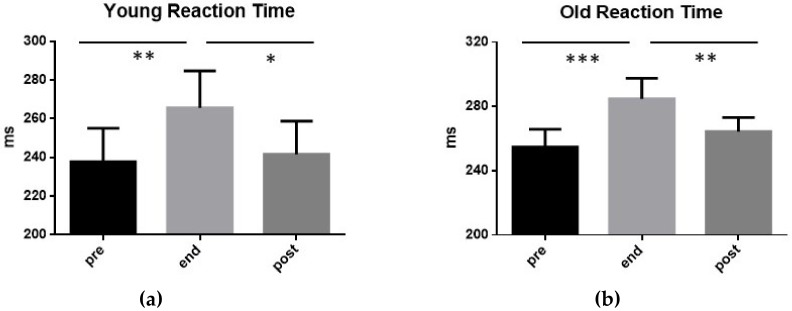
Values of RT exhibited by the 15 subjects of group YOUNG (**a**) and by the 15 subjects of group OLD (**b**) performing an exhaustive exercise. In both cases, RT mean value measured before the exercise (pre), at its conclusion (end), as well as 15 min after its end (post) are displayed. Symbols from ANOVA with Dunn’s multiple comparison test: * *p* < 0.05, ** *p* < 0.01, *** *p* < 0.001.

In Figure 2, it can be seen that, in both YOUNG and OLD groups, the RT augmented significantly at the end of the exhaustive exercise, and returned to the pre-exercise values 15 min after its end. In particular, in the group YOUNG, RT increased from 237.7 ms (±17.40 SD) before the exercise, to 265.6 ms (±19.14 SD) at its end, and returned to pre-exercise values after 15 min (241.6 ms ± 17.15 SD). In the group OLD, RT increased from 254.7 ms (±11.30 SD) before the exercise, to 284.60 ms (±12.80 SD) at its end, and returned to pre-exercise values after 15 min (264.40 ms ± 8.58 SD).

It is interesting to note that the mean values of RT measured in the group YOUNG were significantly inferior to that of the group OLD before the exercise (t-test: *p* < 0.01), at its end (t-test: *p* < 0.01) and 15 min after its completion (t-test: *p* < 0.001).

**Figure 3:**
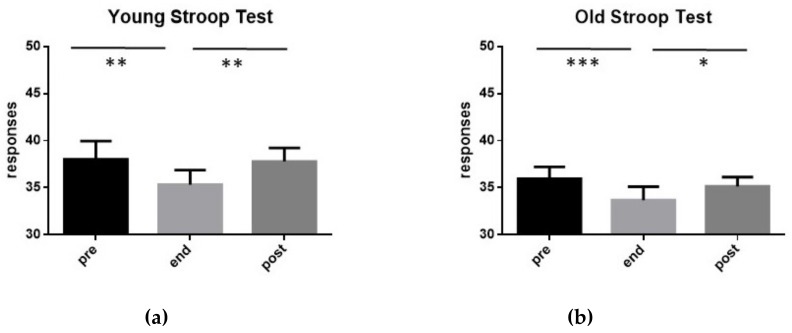
Values of SCWT mean score exhibited by the 15 subjects of group YOUNG (**a**) and by the 15 subjects of group OLD (**b**) performing an exhaustive exercise. In both cases, SCWT mean score measured before the exercise (pre), at its conclusion (end), as well as 15 min after its end (post) are shown. Symbols from ANOVA with Dunn’s multiple comparison test: * *p* < 0.05, ** *p* < 0.01, *** *p* < 0.001.

In Figure 3, it can be observed that, in both YOUNG and OLD groups, the performances at SCWT worsened significantly at the end of the exhaustive exercise, and returned to the pre-exercise values 15 min after its end. In particular, in the group YOUNG, the SCWT mean score decreased from 38.0 (±1.96 SD) before the exercise, to 35.33 (±1.54 SD) at its end, and returned to pre-exercise values after 15 min (37.80 ± 1.41 SD). In the group OLD, SCWT mean score reduced from 38.9 (±1.19 SD) before the exercise, to 33.67 (±1.45 SD) at its end, and returned to pre-exercise values after 15 min (35.13 ± 0.99 SD).

It is worth noting that the mean values of SCWT mean score measured in the group YOUNG were significantly higher than that of the group OLD before the exercise (t-test: *p* < 0.01), at its end (t-test: *p* < 0.01) and 15 min after its completion (t-test: *p* < 0.001).

**Figure 4:**
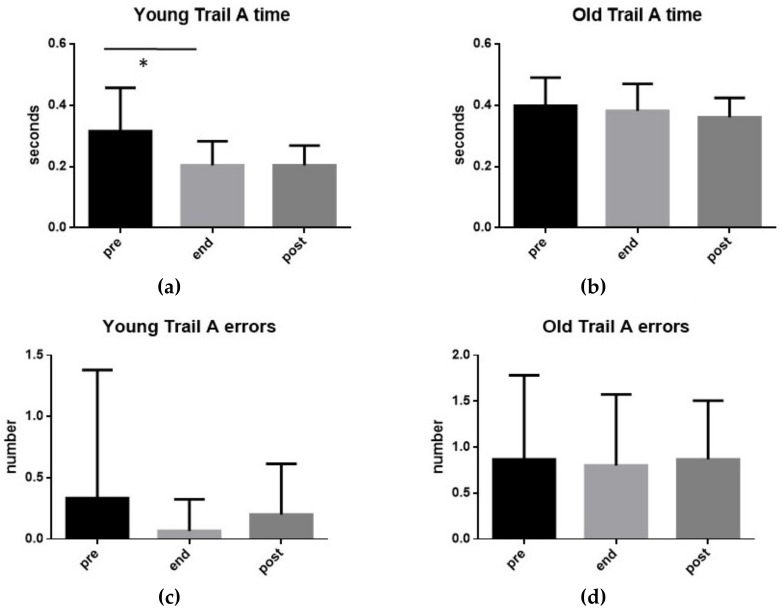
TMT-A. Execution time (**a**,**b**) and number of errors (**c**,**d**) found in the 15 subjects of group YOUNG (**a**,**c**) and in the 15 subjects of group OLD (**b**,**d**) performing an exhaustive exercise. Mean values measured before the exercise (pre), at its conclusion (end), as well as 15 min after its end (post) are shown. Symbols from ANOVA with Dunn’s multiple comparison test: * *p* < 0.05.

In Figure 4, there are shown, for both YOUNG and OLD groups, the performances (time and errors) at TMT-A before the exhaustive exercise (pre), at the conclusion (end) and 15 min after its end (post). As can be seen, the only statistically significant variation (t-test: *p* < 0.05) was observed in the YOUNG group where a reduction of the test execution time was detected at the end of the exercise compared to the pre-exercise values.

It should be noted that the time taken by the YOUNG group for the execution of the TMT-A was significantly lower than for the OLD group, both at the end of the exercise (t-test: *p* < 0.001) and after 15 min prior to its end (t-test: *p* < 0.001). Similarly, the number of errors made by the YOUNG group during the execution of the TMT-A was significantly lower than the OLD group both at the end of the exercise (t-test: *p* < 0.01) and after 15 min prior to its completion (t-test: *p* < 0.01).

**Figure 5:**
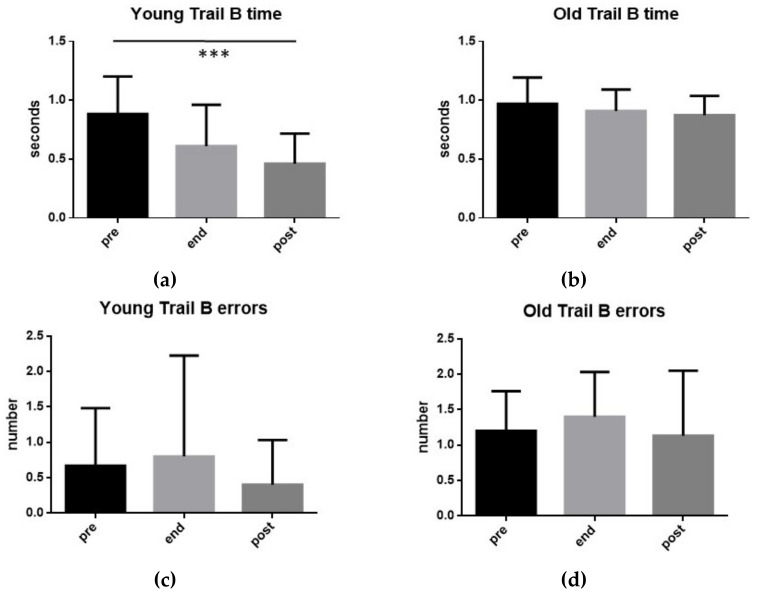
TMT-B. Execution time (**a**,**b**) and number of errors (**c**,**d**) found in the 15 subjects of group YOUNG (**a**,**c**) and in the 15 subjects of group OLD (**b**,**d**) performing an exhaustive exercise. Mean values measured before the exercise (pre), at its conclusion (end), as well as 15 min after its end (post) are shown. Symbols from ANOVA with Dunn’s multiple comparison test: * *p* < 0.001.

Figure 5 shows for both YOUNG and OLD groups, the performances (time and errors) at TMT-B before the exhaustive exercise (pre), at the conclusion (end), and 15 min after its end (post). As can be seen, the only statistically significant variation (t-test: *p* < 0.001) was observed in the YOUNG group where a reduction of the execution time was detected at 15 min after its end of the exercise compared to the pre-exercise values.

It should be noted that the time taken by the YOUNG group for the execution of the TMT-B was significantly lower than the OLD group both at the end of the exercise (t-test: *p* < 0.01) and after 15 min from its end (t-test: *p* < 0.001). Similarly, the number of errors made by the YOUNG group during the execution of the TMT-A was significantly lower than the OLD group both before the exercise (t-test: *p* < 0.01) and during the last 15 min before its end (t-test: *p* < 0.001).

**Figure 6:**
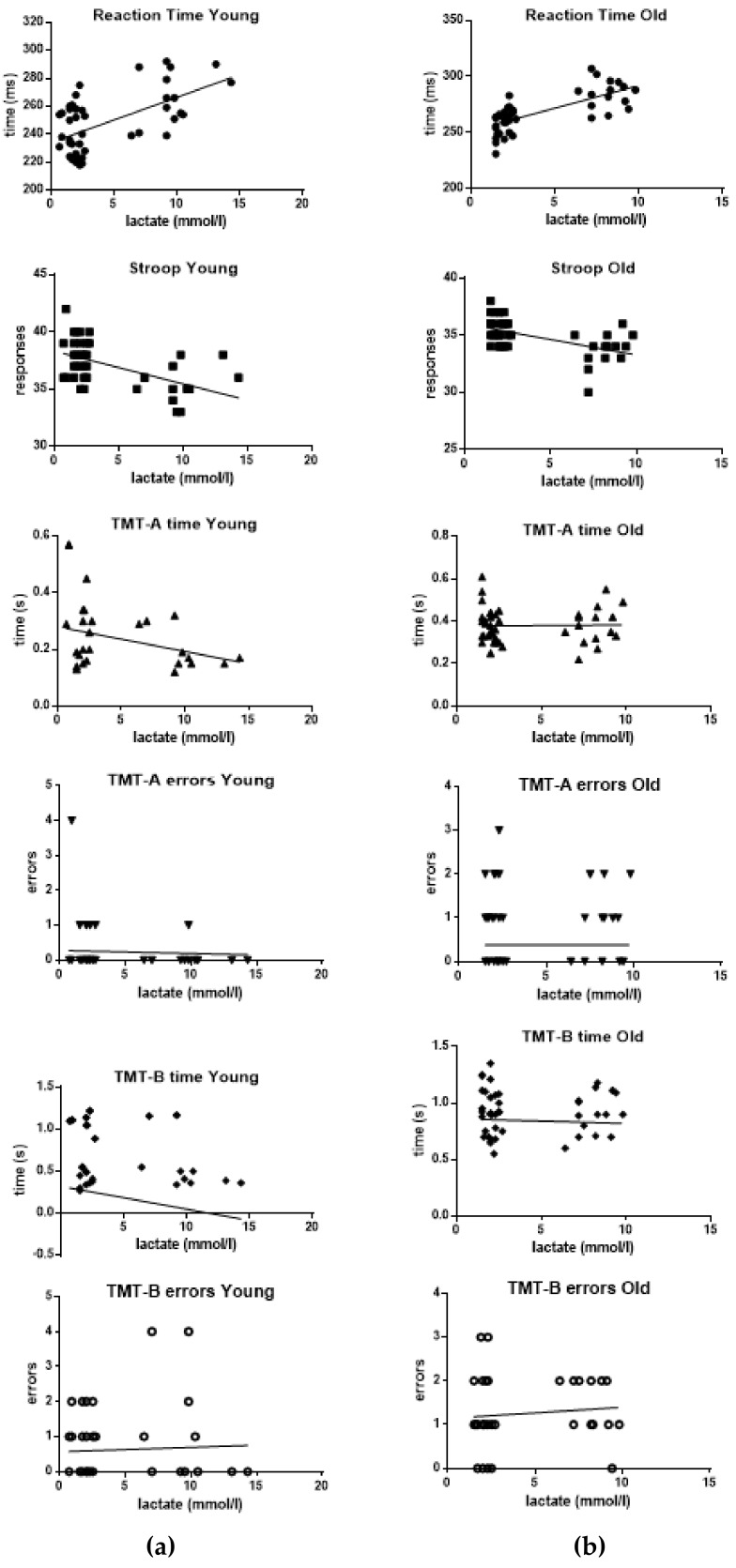
Correlations between blood lactate levels and performances at RT, SCWT, TMT-A and TMT-B in the 15 subjects of group YOUNG (**a**) and in the 15 subjects of group OLD (**b**).

Finally, the correlations between blood lactate levels and the performance of the participants in the various tests were analyzed and results are summarized in Figure 6.

First of all, the statistical analysis showed a strong positive correlation between blood lactate levels and RT values, both in the group YOUNG (R square = 0.3445; *p* < 0.001) and in the group OLD (R square = 0.5288; *p* < 0.001).

A significant negative correlation was also found between blood lactate levels and SCWT mean score, both in the group YOUNG (R square = 0.2963; *p* < 0.001) and in the group OLD (R square = 0.2878; *p* < 0.001).

Regarding the possible correlations between blood lactate levels and TMT-A and TMT-B tests, only a slight negative correlation between lactate and time was found for TMT-A in the group YOUNG (R square = 0.0915; *p* = 0.044). No other statistically significant correlation was detected in both YOUNG and OLD groups.

## 4. Discussion

The results of this study can be summarized as follows:

1) A significant positive correlation was observed between the levels of lactate in the blood and the levels of RT;
2) A significant negative relationship was observed between blood lactate levels and the average SCWT score;
3) No significant correlations between blood lactate levels and TMT scores (time and errors), both A and B were observed;
4) The comparison between the group YOUNG and the group OLD showed that the variations in blood lactate levels, due to exhaustive exercise, and parallel deterioration in the execution of RT and SCWT are significantly more pronounced in the former than in the latter.

The effects of acute physical exercise on the cognitive performances of an adult individual are still under discussion. The existing literature tends to highlight a positive relationship if the exercise is of sub-maximal intensity while the effects seem to be negative for exhaustive exercises [2,3,4,5,6,7,9,10,11,14,15,18,19,20,21,24,27,30].

The present study confirm results previously observed for working memory [20], since the increases in blood lactate levels, deriving from exhaustive exercise, are associated with a worsening of executive functions [34,35,36].

However, in this study, not all the domains that fall within the executive functions were systematically analyzed. In fact, having used only RT, SCWT, and TMT were essentially explored processing speed, cognitive flexibility, resistance to interference from external stimuli, visual attention, and task switching.

Therefore, high levels of blood lactate are associated with a deterioration of processing speed, cognitive flexibility, and resistance to interference, with no significant influences on visual attention and task switching. These results support the idea that the tasks for executive functions may show differential results in different conditions of health and functional stages or levels of physical activity, even in normal subjects. Therefore, it is possible to conclude that, under physiological conditions, executive functions are not cognitive abilities stable over time, but rather are capable of quantitatively changing in relation with psycho-physical modifications.

Why an exhaustive exercise influences only certain executive functions is unclear. One possibility is that the high blood lactate levels, induced by the exercise, may affect some areas of the cortex and not others. In this way, functions supported by the prefrontal cortex, as processing speed [26], cognitive flexibility [34], and resistance to interference [12] seem to be affected, while those supported by more posterior cortical areas, such as visual attention [22] and task switching [29], are not.

This possibility seems to be in agreement with what reported by Sudo et al. [27] which found that exhaustive exercise did not alter the performance in a Go/No-Go task and in a Spatial Delayed-Response task.

Sudo and coworkers [27], in their study, conclude that an exhaustive exercise would be able to influence the executive functions, studied through a Go/No-Go task and in a Spatial Delayed-Response task, for changes in oxygenation of the cerebral cortex and not for the increase in blood lactate; in this way a direct action of lactate on brain tissue is excluded,

The possibility that the effects of exhaustive exercise on cognitive processes may also depend on metabolic, vascular, or thermal phenomena cannot be excluded.

However, a negative effect on attentional processes of high blood lactate levels induced with an intravenous infusion of a lactate solution was found [3] and a negative correlation between CSF lactate levels and cognitive capabilities was observed [37,38,39,40]. Moreover, lactate receptors were found in the brain [16] and a role as a neural regulator for lactate was proposed [23].

Among the limitations of the present study, it is necessary to report the training level of each group of subjects that might influence the final results.

## 5. Conclusions

In conclusion, the present study supports the possibility that high levels of blood lactate induced by an exhaustive exercise could adversely affect the executive functions pertaining to the prefrontal cortex. These influences, even if reduced in quantity, remain present even in older people.

## Figures and Tables

**Table 1:** The anthropometric characteristics of the participants

Subject	YOUNG				OLD			
	Age (years)	Height (cm)	Weight (kg)	BMI *	Age (years)	Height (cm)	Weight (kg)	BMI
1	28	169	71	24.86	60	168	73	25.86
2	24	178	77	24.30	55	171	73	24.96
3	27	168	69	24.45	58	166	70	25.40
4	20	170	71	24.57	65	173	71	23.72
5	29	175	74	24.16	59	178	80	25.25
6	22	174	79	26.09	60	174	78	25.76
7	23	181	83	25.34	58	162	65	24.77
8	28	171	78	26.67	59	174	79	26.09
9	25	166	69	25.04	61	169	72	25.21
10	23	177	80	25.54	57	171	76	25.99
11	21	173	78	26.06	55	171	69	23.60
12	20	170	73	25.26	59	170	73	25.26
13	25	176	74	23.89	60	168	70	24.80
14	27	168	70	24.80	61	167	70	25.10
15	29	173	71	23.72	56	176	72	23.24
**Mean**	**24.73**	**172.60**	**74.47**	**24.98**	**58.87**	**170.53**	**72.73**	**25.00**
SD **	**3.17**	**4.26**	**4.42**	**0.85**	**2.59**	**4.12**	**4.06**	**0.87**

* BMI = Body Mass Index; ** **SD** = Standard Deviation.
